# Molecular identification and whole genome sequence analyses of methicillin-resistant and mastitis-associated *Staphylococcus aureus* sequence types 6 and 2454 isolated from dairy cows

**DOI:** 10.7150/jgen.90833

**Published:** 2024-01-18

**Authors:** Mohammad H. Rahman, Mohamed E. El Zowalaty, Linda Falgenhauer, Mohammad F. R. Khan, Jahangir Alam, Najmun N. Popy, Hossam M. Ashour, Md. Bahanur Rahman

**Affiliations:** 1 Department of Microbiology and Hygiene, Bangladesh Agricultural University, Mymensingh-2202, Bangladesh.; 2 Veterinary Medicine and Food Security Research Group, Medical Laboratory Sciences Program, Faculty of Health Sciences, Abu Dhabi Women's Campus, Higher Colleges of Technology, Abu Dhabi, UAE.; 3 Institute of Hygiene and Environmental Medicine, Justus Liebig University Giessen, Biomedical research center Seltersberg, Schubertstrasse 81, 35392 Giessen, Germany.; 4 National Institute of Biotechnology, Savar, Dhaka, Bangladesh.; 5 Department of Integrative Biology, College of Arts and Sciences, University of South Florida, St. Petersburg, Florida, USA.

## Abstract

The emergence of antimicrobial-resistant and mastitis-associated *Staphylococcus aureus* is of great concern due to the huge economic losses worldwide. Here, we report draft genome sequences of two *Staphylococcus aureus* strains which were isolated from raw milk samples obtained from mastitis-infected cows in Bangladesh. The strains were isolated and identified using conventional microbiological and molecular polymerase chain reaction (PCR) methods. Antibiotic susceptibility testing was performed. Genomic DNA of the two strains was extracted and the strains were sequenced using the Illumina NextSeq 550 platform. The assembled contigs were analyzed for virulence determinants, antimicrobial resistance genes, extra-chromosomal plasmids, and multi-locus sequence type (MLST). The genomes of the two strains were compared with other publicly available genome sequences of *Staphylococcus aureus* strains. The raw read sequences were downloaded and all sequence files were analyzed identically to generate core genome phylogenetic trees. The genome of BR-MHR281strain did not harbour any antibiotic resistance determinants, however BR-MHR220 strain harbored *mecA* and *blaZ* genes. Analysis of BR-MHR220 strain revealed that it was assigned to sequence type (ST-6), clonal complex (CC) 5 and *spa* type t304, while BR-MHR281 strain belonged to ST-2454, CC8, and harbored the *spa* type t7867. The findings of the present study and the genome sequences of BR-MHR220 and BR-MHR281 strains will provide data on the detection and genomic analysis and characterization of mastitis-associated *Staphylococcus aureus* in Bangladesh. In addition, the findings of the present study will serve as reference genomes for future molecular epidemiological studies and will provide significant data which help understand the prevalence, pathogenesis and antimicrobial resistance of mastitis-associated *Staphylococcus aureus*.

## Introduction

Bovine mastitis is a multi-factorial, multi-etiological, highly contagious common livestock production-related disease. Bovine mastitis causes huge economic losses and leads to great implications in dairy industry worldwide due to the reduced milk quality and quantity in dairy herds 1. Mastitis is a very complex and multi-etiological disease caused by more than 140 species of bacteria 2.

*Staphylococcus aureus* (*S. aureus*) is one of the major foodborne pathogens associated with various human infections and animal diseases including important livestock such as cattle, cows, sheep and goats 3. *S. aureus* is the most common etiological agent associated with bovine mastitis worldwide and results in a range of manifestations, including a large proportion of subclinical and chronic infections 4,5. Among livestock, cows are a common reservoir of *S. aureus,* and dairy cattle frequently experience clinical and subclinical mastitis due to *S. aureus* intramammary infections 6.

*S. aureus* possesses an arsenal of virulence and antimicrobial resistance determinants which are subject to horizontal genetic transfer and recombination 7. Genome sequencing has provided insight into the genotypic features of various *S. aureus* clones worldwide, delivering more options for developing therapeutics and molecular diagnostic tools to detect resistant and difficult-to-treat strains.

Genome sequencing and characterization of *S. aureus* isolated from bovine milk is an important tool in the epidemiological studies of bovine mastitis. They can provide clinically relevant results and contribute to the understanding of the pathogen's dissemination and contagious properties 4,8.

The detection of antimicrobial resistant *S. aureus* strains isolated from bovine mastitis, its zoonotic potential, and the possibility of transmission to humans via the consumption of raw unpasteurized dairy and livestock products are increasing public health concerns 9,10. Irrational use of antibiotics in bovine mastitis treatment may results in the development of resistant strains and residual antibiotics in milk also pose serious public health concerns 9,11. The factors, etiologies, treatment, and molecular characterization of common bovine mastitis-causing pathogens were recently reported 12, yet no studies on the application of whole genome sequencing in mastitis-causing pathogens were reported from Bangladesh. Here we report the draft genome sequences of two *S. aureus* strains isolated from raw milk samples obtained from mastitis lactating cows in Bangladesh.

## Materials and Methods

### Ethics statement

The study protocol entitled "Development of polyvalent mastitis vaccine and probiotics for prevention of mastitis in cows" reference number AWEEC/BAU/2020(44) was approved by the Animal Welfare and Experimentation Ethics Committee, Bangladesh Agricultural University, Mymensingh-2202, Bangladesh.

### Sample collection

Milk samples were obtained from 36-month and 46-month-old female lactating Holstein Friesian (*Bos taurus taurus*) cows as previously reported 13. Cow udders were washed with clean water and dried, then the udder teats were rubbed with 70% ethanol. The first two strings were discarded, and California mastitis test was performed to determine the milk somatic cell counts in milk samples as previously reported 13. Milk samples (10 mL) were collected in sterile tubes and samples were transported to the laboratory maintaining a cold chain for further analysis. Somatic cell counting was performed using Lactoscan Combo's SCC (Milkotronic Ltd, Bulgaria) according to the manufacturer's protocol.

### Bacterial isolation

Milk samples (500 µL) were inoculated in 10 mL nutrient broth and incubated at 37°C for 18 hours and subsequently streaked on mannitol salt agar media (HiMedia). The inoculated plates were incubated at 37 °C for 24 hours and sub-cultured to isolate presumptively identified *S. aureus* pure colonies as previously reported 13.

### DNA extraction and bacterial identification using PCR

Presumptive *S. aureus* pure single colonies were confirmed using *S. aureus* primer-specific PCR. Genomic DNA was extracted using genomic DNA Purification Kit (Promega, WI, USA) and isolates were confirmed by PCR using species-specific primers (GCG ATT GAT GGT GAT ACG GTT and AGC CAA GCC TTG ACG AAC TAA AGC) targeting the *nuc* gene as previously reported 14.

### Antimicrobial Sensitivity testing

Antimicrobial susceptibility profiles against ciprofloxacin (5µg), cefoxitin (30µg), chloramphenicol (10µg), doxycycline (30µg), fosfomycin (50µg), gentamicin (10µg), levofloxacin (5µg), sulfamethoxazole - trimethoprim (1.25/23.75µg), and tetracycline (30µg) were determined using the Kirby-Bauer disk diffusion method (Oxoid Ltd., UK), as previously reported. The results were interpreted according to Clinical and Laboratory Standard Institute guidelines 15.

### Whole-genome sequencing analysis

*S. aureus* PCR-confirmed isolates were submitted to Invent Technology Ltd. (Banani, Dhaka, Bangladesh) for whole-genome sequencing as recently reported 13. Sequencing libraries were prepared using a Nextera XT library preparation kit (Illumina Inc., CA, USA) and sequencing was performed on the Illumina NextSeq 550 platform (Illumina Inc., CA, USA) using the high-output reagent kit with 150 nt maximal read length.

### Bioinformatic analyses

Gene predictions and annotations were performed using the National Center for Biotechnology Information (NCBI) Prokaryotic Genome Annotation Pipeline (PGAP) 16. Raw paired-end reads were quality checked and assembled to contigs using the ASA^3^P pipeline 17. The detection of antibiotic resistance genes was performed using Resfinder 4.0 18. SCCmec type determination was performed using the SCCmecFinder tool, (https://cge.food.dtu.dk/services/SCCmecFinder/). Multilocus sequence type determination was performed using PubMLST 19. *spa* type determination was performed using spaTyper 1.0 20. Virulence gene determination was performed using VFanalyzer 21. The presence of Cap5A-P proteins from *S. aureus* USA300_FPR3757 22 was determined using tblastN. Core-genome-based analysis was performed using ParSNP of the Harvest Suite package 23. The resulting trees were annotated using ITol v 6.6 24.

## Results and Discussion

In the present study, a total of 423 randomly selected lactating cows were tested for the detection of *S. aureus* in their milk samples. It was found that 44.68% (189/423) of the cows were mastitis positive, of which 17.49% (74/423) were clinical and 27.19% (115/423) sub-clinical mastitis. Isolation of *S. aureus* was performed using mannitol salt agar which were subsequently confirmed using *nuc*-gene specific PCR. It was found that 54.49% (103 out of 189 mastitis affected cows) were infected with *Staphylococcus aureus*.

Genome sequences of two *S. aureus* BR-MHR220 and BR-MHR281 strains were generated and raw paired-end reads (average read count 18,863,133; average coverage 1024×, average read length 135 nt) were quality checked and assembled to contigs using the ASA^3^P pipeline 17. Assembly was performed using SPAdes v3.13.0 25 integrated in ASA^3^P. Contigs smaller than 200 bp were discarded. Contigs were uploaded to NCBI and annotated using the NCBI Prokaryotic Genome Annotation Pipeline v6.0 16.

For BR-MHR220 genome, a total number of 54 contigs and 2,815,914 bp, with a G+C content of 32.75%, and a *N_50_* value of 321,760 bp was achieved. For BR-MHR281 genome, a total number of 28 contigs and 2,728,146 bp, with a G+C content of 32.73%, and a *N_50_* value of 470,470 bp was achieved.

Gene predictions and annotations identified 2,788 coding DNA sequences (CDS), 54 tRNAs, 4 ncRNA and 4 rRNA genes for BR-MHR220, and 2,677 coding DNA sequences, 56 tRNAs, 4 ncRNA and 4 rRNA genes for BR-MHR281.

Phenotypic antimicrobial profiling revealed that both BR-MHR220 and BR-MHR281 strains were resistant to cefoxitin, methicillin, and oxacillin. Additionally, BR-MHR220 strain was resistant to linezolid and penicillin G, while BR-MHR281 strain was resistant to gentamicin and levofloxacin. Both BR-MHR220 and BR-MHR281 strains were sensitive to chloramphenicol, ciprofloxacin, doxycycline, and sulfamethoxazole-trimethoprim. Additionally, BR-MHR220 strain was sensitive to gentamicin and levofloxacin, while BR-MHR281 strain was sensitive to linezolid, penicillin G, and tetracycline.

Genome analyses of the two BR-MHR220 and BR-MHR281 strains were performed and no antibiotic resistance genes were detected in strain BR-MHR281, however strain BR-MHR220 harbored the *mecA* and *blaZ* genes. The* mecA* gene is presumably located on a SCCmec type IVa(2B) element. The reported genotype did not correlate with the phenotypic resistance detected. The discrepancy between genotypic and phenotypic resistance may be explained by possible mutations of porin genes or overexpression of efflux pumps.

Only few *S. aureus* STs were previously reported to be positive for *blaZ* or *mecA*. Some human-adapted lineages, such as ST-5 (CC5), ST-8 (CC8), and their variants were previously isolated from cows and were positive for *blaZ* and *mecA* 26-28*.* In the present study, we reported the detection of ST-6 (CC5) isolate positive for both* blaZ* and *mecA.*

Determination of virulence genes was performed using VFanalyzer 20. In addition, the presence of Cap5A-P proteins from *S. aureus* USA300_FPR3757 21 in both BR-MHR220 and BR-MHR281 genomes was determined using tblastN. The overview of detected virulence genes is depicted in Supplementary Table 1. Homologues of 60 virulence genes were detected in both isolates, while specific 19 and 13 virulence genes were only detected in BR-MHR220 and BR-MHR281, respectively. The virulence genes detected only in BR-MHR220 were the cell wall associated fibronectin binding protein *ebh*, the collagen adhesion *cna*, the intracellular adhesin *icaD*, two Ser-Asp rich fibrinogen-binding proteins (*sdrD, sdrE*), six serine proteases (*splA, splB, splC, splD, splE, splF*), Staphylokinase (*sak*), SCIN (*scn*), Enterotoxin A (*sea*), four Exotoxins (set7, set15, set16, set25) and Leukotoxin D (*lukD*). BR-MHR281 harbored seven enterotoxins (*seg, yent2, selk, selm, seln, selo, selq*). All were different from the one found in BR-MHR220. Five exotoxins were detected only in BR-MHR281 (*set17, set21, set26, set30, set39*). The toxic shock syndrome toxin (*tsst*) was detected only in BR-MHR281.

Multilocus sequence type determination was performed using PubMLST 18. *spa* type determination was performed using spaTyper 1.0 19. BR-MHR220 was assigned to sequence type (ST-6), clonal complex (CC) 5 and *spa* type t304, while BR-MHR281 belonged to ST-2454 and CC8 and harbored the *spa* type t7867.

Several bovine-adapted *S. aureus* lineages including CC97, CC133, and CC151 and human-adapted lineages including CC1, CC5, CC8, CC30, and CC45 were previously reported 29. In the present study, two strains belonging to CC5 and CC8 *S. aureus* lineages were isolated from bovine mastitis samples. Similarly, the genome sequences of three human-adapted isolates (two from CC97 and one from CC8), isolated from bovine mastitis samples were previously reported 30. Altogether, the results provide significant insights on the role of genomic characteristics in early *S. aureus* host spillover events and the subsequent adaption to a new host. The zooanthroponotic transfer and the spillover transmission of CC5 and CC8 from humans to bovine in the present study are possible events that require further investigation and comparative genomic analysis.

**Figure 1:**
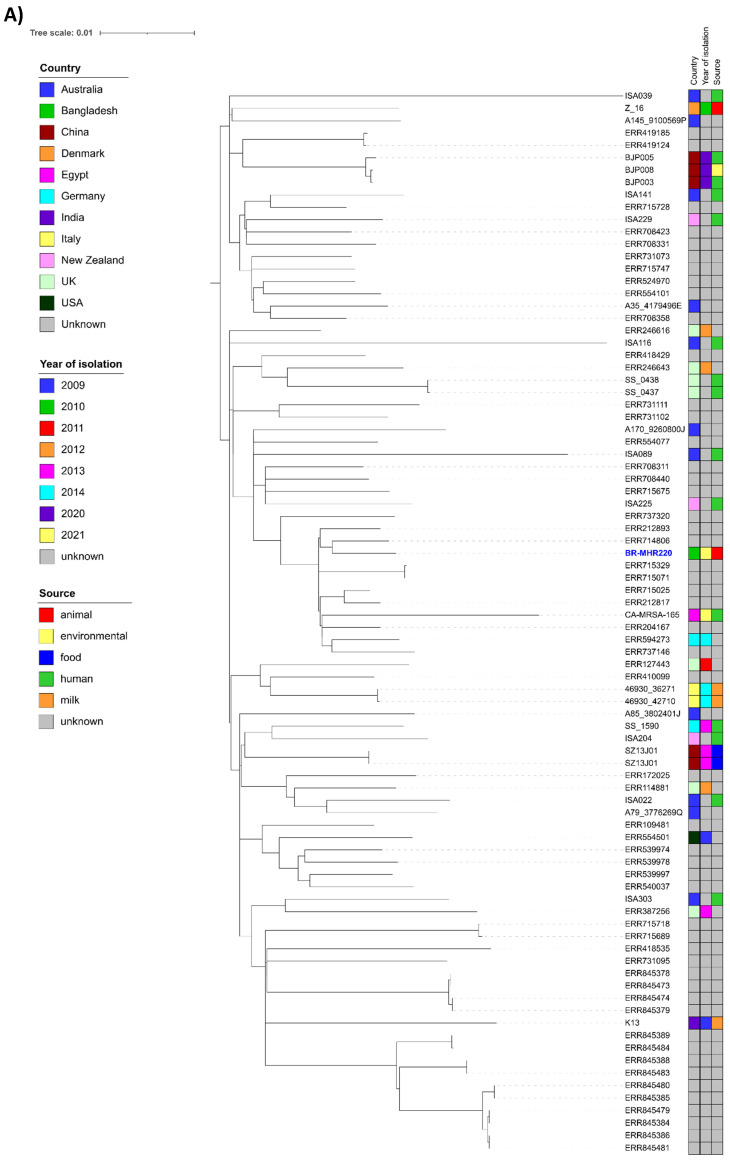

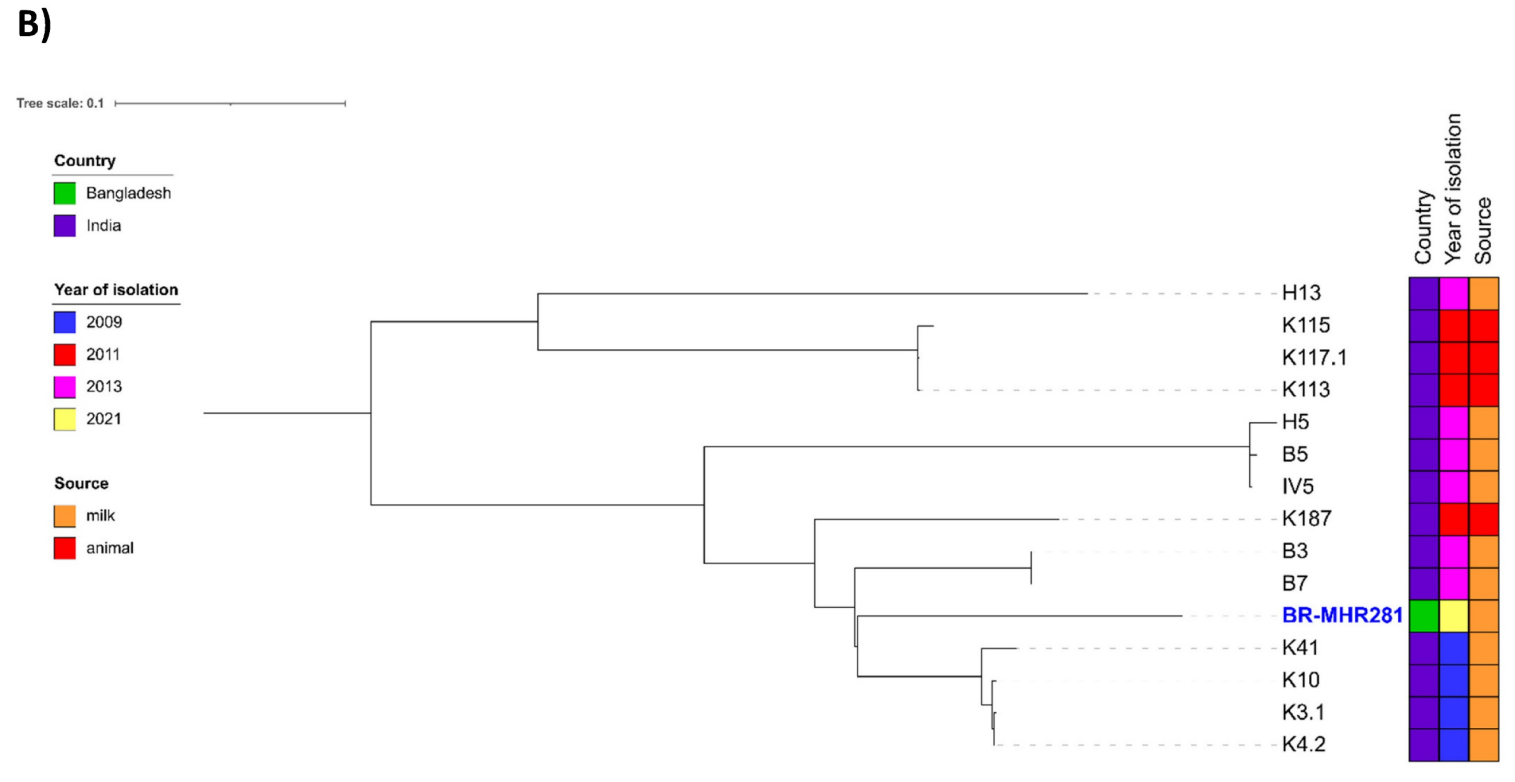
Phylogenetic trees of *S. aureus* strain (A) BR-MHR220 and (B) BR-MHR281. For the core genome-based comparison, ST-6 or ST-2454 sequence information present in PubMLST was used. Trees were annotated using ITOL v. 6.6 and modified using Inkscape.

For comparison of BR-MHR220 and BR-MHR281 with global isolates, ST-6 (n=85, Supplementary Table 2) and ST-2454 (n= 14, Supplementary Table 3) isolate contigs were downloaded from the PubMLST database (as of 23^rd^ January 2023). Core-genome-based analysis was performed using ParSNP of the Harvest Suite package 22. The resulting trees were annotated using ITol v 6.6 23. As depicted in Figure 1A, the closest relative to BR-MHR220 was ERR714806, an isolate collected in the frame of a study on MRSA in England (source unknown) 31. The closest relative to BR-MHR281 (Figure 1B) was K4.2, isolated from cow milk in India in 2009. The current study describes the use of whole genome sequencing (WGS) methods in the detection of bovine mastitis-associated *S. aureus* isolates in dairy cows in Bangladesh. The findings corroborate the worldwide distribution of *S. aureus* CC8 and CC5 isolates in different host species. At present however, WGS still remains unaffordable and inaccessible tool in resource-limited settings. The genome sequences of *S. aureus* strains in the present study will contribute to advanced understanding of the virulence, host adaptation, zoonotic and zooanthroponotic potential of *S. aureus*.

### Data availability

This whole-genome sequencing project has been deposited at DDBJ/ENA/GenBank under the BioProject number PRJNA716986 (BioSample accession numbers SAMN26025965 and SAMN26025969 and GenBank accession numbers JALBGM000000000 and JALBGI000000000. The versions described in this paper are the first version. The sequences have been submitted to the Sequence Read Archive (SRA) under the accession numbers SRR18182112 and SRR18182108. All isolates used in this study were submitted to Public Databases for molecular typing and microbial genome diversity for curation (https://pubmlst.org/organisms/staphylococcus-aureus) and are publicly available under PubMLST ID numbers 38059 and 38060.

## Supplementary Material

Supplementary tables.
