# Supplementary material for: Molecular identification and whole genome sequence analyses of methicillin-resistant and mastitis-associated Staphylococcus aureus sequence types 6 and 2454 isolated from dairy cows
Source: J Genomics. 2024 Jan 18;12:19–25. doi: 10.7150/jgen.90833 (PMC10845239; doi:10.7150/jgen.90833)
Supplement: Supplementary file 1 — Supplementary tables. [file jgenv12p0019s1.pdf]

Supplementary_Table_1

	A	B	C	D	E
1	SupplementaryTable_1_Virulence				
2	**Virulence factor class**	**Virulence factors**	**Related genes**	**BR-MHR220**	**BR-MHR281**
3					
4	Adherence	Autolysin	atl	+	+
5		Cell wall associated fibronectin binding protein	ebh	+	-
6		Collagen adhesion	cna	+	-
7		Elastin binding protein	ebp	+	+
8		Fibrinogen binding protein	efb	+	+
9		Fibronectin binding proteins	fnbA	+	+
10			fnbB	+	+
11		Intercellular adhesin	icaA	+	+
12			icaB	+	+
13			icaC	+	+
14			icaD	+	-
15			icaR	+	+
16		Ser-Asp rich fibrinogen-binding proteins	sdrC	+	+
17			sdrD	+	-
18			sdrE	+	-
19		Staphylococcal protein A	spa	+	+
20	Enzyme	Cysteine protease	sspB	+	+
21			sspC	+	+
22		Hyaluronate lyase	hysA	+	+
23		Lipase	geh	+	+
24			lip	+	+
25		Serine V8 protease	sspA	+	+
26		Serine protease	splA	+	-
27			splB	+	-
28			splC	+	-
29			splD	+	-
30			splE	+	-
31			splF	+	-
32		Staphylocoagulase	coa	+	+
33		Staphylokinase	sak	+	-
34		Thermonuclease	nuc	+	+
35	Immune evasion	AdsA	adsA	+	+
36		Capsule	capA	+	+
37			capB	+	+
38			capC	+	+
39			capD	+	+
40			capE	+	+
41			capF	+	+
42			capG	+	+
43			capH	+ (cap8 type)	+ (cap5 type)
44			capI	+ (cap8 type)	+ (cap5 type)
45			capJ	+ (cap8 type)	+ (cap5 type)
46			capK	+ (cap8 type)	+ (cap5 type)
47			capL	+	+
48			capM	+	+
49			capN	+	+
50			capO	+	+
51			capP	+	+
52		SCIN	scn	+	-
53		Sbi	sbi	+	+

	A	B	C	D	E
2<br>3	**Virulence factor class**	**Virulence factors**	**Related genes**	**BR-MHR220**	**BR-MHR281**
54	Secretion system	Type VII secretion system	esaA	+	+
55			esaB	+	+
56			esaD	+	+
57			esaE	+	+
58			esaG	+ (8 homologous copies)	+ (2 homologous copies)
59			essA	+	+
60			essB	+	+
61			essC	+	+
62			esxA	+	+
63			esxB	+	+
64			esxC	+	+
65			esxD	+	+
66	Toxin	Alpha hemolysin	hly/hla	+	+
67		Delta hemolysin	hld	+	+
68		Enterotoxin A	sea	+	-
69		Enterotoxin G	seg	-	+
70		Enterotoxin Yent2	yent2	-	+
71		Enterotoxin-like K	selk	-	+
72		Enterotoxin-like M	selm	-	+
73		Enterotoxin-like N	seln	-	+
74		Enterotoxin-like O	selo	-	+
75		Enterotoxin-like Q	selq	-	+
76		Exotoxin	set7	+	-
77			set13	+	+
78			set15	+	-
79			set16	+	-
80			set17	-	+
81			set18	+	+
82			set19	+	+
83			set21	-	+
84			set22	+	+
85			set25	+	-
86			set26	-	+
87			set30	-	+
88			set34	+	+
89			set37	+	+
90			set39	-	+
91		Gamma hemolysin	hlgA	+ (2 homologous copies)	+
92			hlgB	+	+
93			hlgC	+	+
94		Leukotoxin D	lukD	+	-
95		Toxic shock syndrome toxin	tsst	-	+

Supplementary_Table_2

pubMLST id	isolate	country	year	source
6353	ERR127443	UK	2011	unknown
9667	ERR204167	Unknown	unknown	unknown
10154	ERR212893	Unknown	unknown	unknown
10750	ERR172025	Unknown	unknown	unknown
12363	ERR114881	UK	2012	unknown
13401	ERR212817	Unknown	unknown	unknown
14458	ERR246616	UK	2012	unknown
14481	ERR246643	UK	2012	unknown
14791	ERR109481	Unknown	unknown	unknown
18582	ERR387256	UK	2013	unknown
19110	ERR418535	Unknown	unknown	unknown
19220	ERR418429	Unknown	unknown	unknown
19360	ERR410099	Unknown	unknown	unknown
20032	ERR419185	Unknown	unknown	unknown
21734	ERR419124	Unknown	unknown	unknown
24148	ERR554077	Unknown	unknown	unknown
24539	ERR554101	Unknown	unknown	unknown
25493	ERR554501	USA	2009	unknown
25723	ERR708358	Unknown	unknown	unknown
26487	ERR524970	Unknown	unknown	unknown
26622	ERR539974	Unknown	unknown	unknown
26624	ERR539978	Unknown	unknown	unknown
26641	ERR539997	Unknown	unknown	unknown
26661	ERR540037	Unknown	unknown	unknown
27440	ERR708331	Unknown	unknown	unknown
27606	ERR715329	Unknown	unknown	unknown
27761	ERR737146	Unknown	unknown	unknown
28155	ERR715747	Unknown	unknown	unknown
28171	ERR708311	Unknown	unknown	unknown
28207	ERR715071	Unknown	unknown	unknown
29936	ERR715718	Unknown	unknown	unknown
29937	ERR715689	Unknown	unknown	unknown
29946	ERR737320	Unknown	unknown	unknown
29951	ERR708423	Unknown	unknown	unknown
29972	ERR715728	Unknown	unknown	unknown
29979	ERR731095	Unknown	unknown	unknown
30019	ERR715025	Unknown	unknown	unknown
30269	ERR708440	Unknown	unknown	unknown
30316	ERR714806	Unknown	unknown	unknown
30344	ERR715675	Unknown	unknown	unknown
30431	ERR731073	Unknown	unknown	unknown
30445	ERR731102	Unknown	unknown	unknown
30448	ERR731111	Unknown	unknown	unknown
31106	ERR594273	Germany	2014	unknown
31134	ERR845378	Unknown	unknown	unknown
31135	ERR845379	Unknown	unknown	unknown

pubMLST id	isolate	country	year	source
31140	ERR845384	Unknown	unknown	unknown
31141	ERR845385	Unknown	unknown	unknown
31142	ERR845386	Unknown	unknown	unknown
31144	ERR845388	Unknown	unknown	unknown
31145	ERR845389	Unknown	unknown	unknown
31178	ERR845473	Unknown	unknown	unknown
31179	ERR845474	Unknown	unknown	unknown
31184	ERR845479	Unknown	unknown	unknown
31185	ERR845480	Unknown	unknown	unknown
31186	ERR845481	Unknown	unknown	unknown
31188	ERR845483	Unknown	unknown	unknown
31189	ERR845484	Unknown	unknown	unknown
36163	A145_9100569P	Australia	unknown	unknown
36190	A170_9260800J	Australia	unknown	unknown
36216	A35_4179496E	Australia	unknown	unknown
36264	A79_3776269Q	Australia	unknown	unknown
36271	A85_3802401J	Australia	unknown	unknown
36948	SZ13J01	China	2013	food
36970	SZ13J01	China	2013	food
37479	BJP003	China	2020	faeces
37480	BJP005	China	2020	faeces
37481	BJP008	China	2020	other
37671	ISA022	Australia	unknown	unknown
37687	ISA039	Australia	unknown	unknown
37736	ISA089	Australia	unknown	unknown
37763	ISA116	Australia	unknown	unknown
37786	ISA141	Australia	unknown	unknown
37836	ISA204	New Zealand	unknown	unknown
37857	ISA225	New Zealand	unknown	unknown
37861	ISA229	New Zealand	unknown	unknown
37934	ISA303	Australia	unknown	unknown
39485	SS_0437	UK	unknown	unknown
39486	SS_0438	UK	unknown	unknown
41897	SS_1590	Germany	2013	medical device
42113	CA-MRSA-165	Egypt	2021	unknown
42317	Z_16	Denmark	2010	animal
43030	46930_42710	Italy	2014	milk
43034	46930_36271	Italy	2014	milk
43098	K13	India	2009	milk

Supplementary_Table_3

pubMLST id	isolate	aliases	country	year	source
43069	B3	ss3600	India	2013	milk
43070	B5	ss3601	India	2013	milk
43071	B7	ss3602	India	2013	milk
43072	H5	ss3603	India	2013	milk
43073	H13	ss3604	India	2013	milk
43074	IV5	ss3605	India	2013	milk
43076	K3.1	ss3607	India	2009	milk
43078	K4.2	ss3609	India	2009	milk
43080	K113	ss3611	India	2011	animal
43081	K115	ss3612	India	2011	animal
43082	K117.1	ss3613	India	2011	animal
43091	K187	ss3622	India	2011	animal
43097	K10	ss3628	India	2009	milk
43100	K41	ss3631	India	2009	milk
